# In Vitro Activities and Inoculum Effects of Ceftazidime-Avibactam and Aztreonam-Avibactam against Carbapenem-Resistant *Enterobacterales* Isolates from South Korea

**DOI:** 10.3390/antibiotics9120912

**Published:** 2020-12-15

**Authors:** Taeeun Kim, Seung Cheol Lee, Moonsuk Bae, Heungsup Sung, Mi-Na Kim, Jiwon Jung, Min Jae Kim, Sung-Han Kim, Sang-Oh Lee, Sang-Ho Choi, Yang Soo Kim, Yong Pil Chong

**Affiliations:** 1 Division of Infectious Diseases, Department of Medicine, Nowon Eulji University Hospital, Seoul 01830, Korea; sleepju@naver.com; 2 Department of Infectious Diseases, Asan Medical Center, University of Ulsan College of Medicine, Seoul 05505, Korea; sclee628@naver.com (S.C.L.); carukeion@gmail.com (M.B.); trueblue27@naver.com (J.J.); nahani99@gmail.com (M.J.K.); kimsunghanmd@hotmail.com (S.-H.K.); soleemd@amc.seoul.kr (S.-O.L.); sangho@amc.seoul.kr (S.-H.C.); yskim@amc.seoul.kr (Y.S.K.); 3 Department of Laboratory Medicine, Asan Medical Center, University of Ulsan College of Medicine, Seoul 05505, Korea; sung@amc.seoul.kr (H.S.); mnkim@amc.seoul.kr (M.-N.K.)

**Keywords:** carbapenem-resistant *Enterobacterales*, ceftazidime-avibactam, aztreonam-avibactam, inoculum effect, susceptibility

## Abstract

Ceftazidime-avibactam (CAZ-AVI) and aztreonam-avibactam (AZT-AVI) are novel antibiotic combinations active against multidrug-resistant Gram-negative pathogens. This study aimed to evaluate their in vitro activities and inoculum effects in carbapenem-resistant *Enterobacterales* (CRE), including carbapenemase-producing (CP)-CRE and non-CP-CRE. A total of 81 independent clinical isolates of carbapenem-resistant *Escherichia coli* and *Klebsiella pneumoniae* were collected. CAZ-AVI and AZT-AVI minimal inhibitory concentrations (MICs) were evaluated by broth microdilution using standard and high inocula. The inoculum effect was defined as an ≥8-fold increase in MIC with high inoculum. Phenotypic determination of β-lactam resistance mechanism and PCR for carbapenemase genes were performed. Of the 81 CRE isolates, 35 (43%) were CP-CRE. Overall, 73% of the isolates were susceptible to CAZ-AVI, and 95% had low AZT-AVI MICs (≤8 µg/mL). The MIC_50/_MIC_90_s of CAZ-AVI and AZT-AVI were 4/≥512 µg/mL and 0.5/4 µg/mL, respectively. CAZ-AVI was more active against non-CP-CRE than against CP-CRE (susceptibility 80% vs. 63%, *p* = 0.08; MIC_50_/MIC_90_, 2/16 μg/mL vs. 4/≥512 μg/mL), whereas AZT-AVI was more active against CP-CRE (MIC_50_/MIC_90_, 0.25/1 μg/mL vs. 0.5/8 μg/mL). All four isolates with high AZT-AVI MIC (≥16 μg/mL) were resistant to CAZ-AVI, but only 18% (4/22) of CAZ-AVI-resistant isolates had high AZT-AVI MIC. The rates of the inoculum effect for CAZ-AVI and AZT-AVI were 18% and 47%, respectively (*p* < 0.001). Interestingly, the frequency of the AZT-AVI inoculum effect was higher in *K. pneumoniae* than *E. coli* (64% vs. 8%, *p* < 0.001). AZT-AVI is more active against CRE than CAZ-AVI, even in CP-CRE and CAZ-AVI-resistant isolates. The presence of a substantial inoculum effect may contribute to clinical failure in high-inoculum infections treated with AZT-AVI.

## 1. Introduction

Carbapenem-resistant *Enterobacterales* (CRE) challenge pharmaceutical chemists and clinicians on account of their difficult-to-treat resistance and increasing global prevalence [1]. Due to the limited therapeutic options for CRE infections, the polymyxins (colistin and polymyxin B) are frequently used as last resort drugs. However, their high rates of nephrotoxicity, which range from 30% to 60%, make their use problematic [2]. Avibactam, a new non-β-lactam β-lactamase inhibitor, is an inhibitor of class A β-lactamases, including extended-spectrum β-lactamases (ESBLs) and *Klebsiella pneumoniae* carbapenemases (KPCs), and also class C (AmpC) and some class D (OXA-48) β-lactamases [3]. Ceftazidime-avibactam shows promising activity against CRE strains, such as KPC-producing *K. pneumoniae* and *Escherichia coli* [4]. However, ceftazidime-avibactam is usually not active against class B metallo-β-lactamase (MBL)-producing CRE [4]. After the introduction of ceftazidime-avibactam into clinical use, cases of resistance due to various mechanisms have been increasingly reported [5].

Aztreonam, a monobactam, is unique among currently used β-lactams, in that it is stable to hydrolysis by MBLs [6]. However, it is easily inactivated by ESBLs, AmpC, and KPCs. When combined with avibactam, aztreonam can inhibit cell wall synthesis in MBL-producing bacteria, despite the presence of co-carried β-lactamases such as ESBLs and AmpC [7]. Thus, aztreonam-avibactam can be more effective than ceftazidime-avibactam against MBL-producing strains. However, there are limited data on the susceptibility of aztreonam-avibactam to CRE. In addition, β-lactam antibiotics, especially β-lactam/β-lactamase inhibitors, are known to display an inoculum effect of variable severity against Gram-negative bacteria. The inoculum effect is a laboratory phenomenon described as a significant increase in the minimal inhibitory concentration (MIC) of an antibiotic when the number of bacteria inoculated increases [8]. Reduced in vitro activity of the β-lactams against a dense bacterial population is commonly attributed to the presence of a high level of β-lactamase. In a clinical situation involving a high bacterial burden, such as an abscess, the inoculum effect may lead to treatment failure.

This study aimed to compare the in vitro activities of ceftazidime-avibactam and aztreonam-avibactam and their inoculum effects in carbapenemase-producing (CP-CRE) and non-CP-CRE isolates. It also examined the relationship of their in vitro activities and inoculum effects to the carbapenem resistance mechanisms of the target bacteria.

## 2. Materials and Methods

### 2.1. Bacterial Isolates and Study Design

Carbapenem-resistant *E. coli* and *K. pneumoniae* isolates were collected from consecutive patients who had no prior exposure to ceftazidime-avibactam or aztreonam-avibactam at Asan Medical Center, a 2700-bed tertiary care center in Seoul, South Korea. A total of 81 carbapenem-resistant *E. coli* and *K. pneumoniae* isolates (25 and 56 isolates, respectively) were collected from January 2014 to October 2018. Only the first CRE isolate from each patient was included in the study. Species identification and initial antimicrobial susceptibility testing was performed with a MicroScan Walk-Away plus System using a Neg Combo Panel Type 72 (Dade Behring Inc., West Sacramento, CA, USA). CRE isolates, defined as those resistant to meropenem or imipenem (MIC ≥ 4 μg/mL) [9,10], were stored at −80 °C. They were streaked on an agar plates and incubated 24 h before experiments. The composition of the study isolates by specimen source was as follows: blood stream, n = 57 (70.4% of all isolates tested), intra-abdominal, n = 9 (11.1%), urinary tract, n = 7 (8.6%), respiratory tract, n = 5 (6.2%), and soft tissue, n = 3 (3.7%).

### 2.2. Antimicrobial Susceptibility Testing and the Inoculum Effect

Antimicrobial susceptibility testing for ceftazidime, aztreonam, ceftazidime-avibactam, aztreonam-avibactam, meropenem, colistin, and tigecycline was performed in triplicate using standard broth microdilution [9,11]. Avibactam was tested at a fixed concentration of 4 μg/mL. MICs were interpreted according to the Clinical and Laboratory Standards Institute (CLSI) breakpoints for all antimicrobial agents except for those for which CLSI breakpoints are not yet available [9]: aztreonam-avibactam, for which clinical breakpoints have not yet been assigned and tigecycline and colistin, for which the European Committee on Antimicrobial Susceptibility Testing (EUCAST) MIC breakpoints were applied [12]. To determine whether there was an inoculum effect with ceftazidime-avibactam, aztreonam-avibactam, and meropenem, the MICs of each antibiotic with high inocula (1 × 10^7^ CFU/mL) were compared to those with standard inocula (1 × 10^5^ CFU/mL) [13,14]. Our definition of an inoculum effect was an 8-fold or greater MIC increase in testing with the high inoculum [8,15]. As MIC values > 256 μg/mL for ceftazidime-avibactam were not further examined, the presence of the inoculum effect for such isolates was not determined. *E. coli* ATCC 25922 and *K. pneumoniae* ATCC 700603 were used as quality control strains for each test. All results determined with these strains were within the CLSI quality control ranges. Ceftazidime, aztreonam, meropenem, tigecycline, and colistin were purchased from Sigma-Aldrich (St. Louis, MO, USA) and avibactam was obtained from AdooQ Bioscience (Irvine, CA, USA). 

### 2.3. Basis of Resistance and Molecular Identification of β-Lactamase Genes

The carbapenem resistance mechanisms of each isolate were examined to determine the impact of resistance mechanisms on antimicrobial susceptibility patterns and the inoculum effect. The modified carbapenem inactivation method, with high sensitivity and specificity, was used to confirm carbapenemase production in all the study CRE isolates [16]. For the carbapenemase-producing (CP) isolates, PCR was used to amplify carbapenemase genes (*bla*_KPC_, *bla*_IMP_*, bla*_VIM_, *bla*_NDM_, and *bla*_OXA-48-like)_ according to the procedures described in previous studies [17,18,19]. Non-carbapenemase-producing (non-CP) isolates usually acquire carbapenem resistance by the production of extended-spectrum β-lactamase (ESBL) and/or AmpC cephalosporinase (AmpC) in conjunction with membrane impermeability or active drug efflux. To identify the β-lactamase types among the non-CP-CRE, the presence of ESBL was determined by the MicroScan ESBL detection test (included in Neg Combo Panel Type 72) using cefotaxime and ceftazidime alone and in combination with clavulanic acid. For isolates not confirmed by the MicroScan ESBL detection test, the double-disk synergy test was performed in addition, using cefotaxime (30 μg), ceftazidime (30 μg), cefepime (30 μg), and amoxicillin plus clavulanate (20 μg and 10 μg each) disks [20,21]. As non-susceptibility to cefoxitin (MIC >8 μg/mL) is considered a surrogate marker for the presence of high-level production of AmpC, isolates non-susceptible to cefoxitin were further characterized by the AmpC confirmatory test using cefoxitin and cloxacillin [22,23]. Cefepime, ceftazidime, cefotaxime, and amoxicillin-clavulanic acid disk were purchased from Bio-rad (Hercules, CA, USA), and cefoxitin disks were obtained from Oxoid (Basingstoke, UK). 

### 2.4. Statistical Analysis

Differences between groups were analyzed using the χ^2^ test or Fisher’s exact test as appropriate. A two-sided *p* < 0.05 was considered statistically significant. SPSS version 24.0 (IBM, Armonk, NY, USA) was used in the statistical analyses.

## 3. Results

Out of the 81 CRE isolates collected, 25 (31%) were *E. coli,* and 56 (69%) were *K. pneumoniae.* Of these 81 isolates, 35 (43%) were CP-CRE; they consisted of 7 *E. coli* and 28 *K. pneumoniae* isolates, of which 17 had KPC and 11 had New Delhi Metallo-β-lactamase (NDM). Among ceftazidime-avibactam, aztreonam-avibactam, and the comparator antimicrobial agents, aztreonam-avibactam had an overall MIC_50_/MIC_90_ of 0.5/4 μg/mL and was the antimicrobial with the highest activity against the CRE isolates (Supplemental Table S1). Since breakpoint criteria have not been defined for aztreonam-avibactam, we stratified the aztreonam-avibactam MICs of isolates as low (≤8 µg/mL) vs. high MIC (≥16 µg/mL). Ninety-five percent of the isolates had low aztreonam-avibactam MICs. The percentage susceptibilities to ceftazidime-avibactam, colistin, and tigecycline were 73% (MIC_50_/MIC_90_, 4/≥512 μg/mL), 86% (MIC_50_/MIC_90_, 0.5/8 μg/mL), and 25% (MIC_50_/MIC_90_, 2/8 μg/mL), respectively. Comparison of in vitro antimicrobial susceptibilities of *E. coli* and *K. pneumoniae* strains is shown in Table 1. Most of the tigecycline-resistant isolates and colistin-resistant isolates were *K. pneumoniae.* For both ceftazidime-avibactam and aztreonam-avibactam, the *K. pneumoniae* isolates tended to have lower MICs than the *E. coli* isolates.

Whether a strain was resistant to colistin or tigecycline did not affect its susceptibilities to ceftazidime-avibactam and aztreonam-avibactam (Supplemental Table S2). Aztreonam-avibactam was active against over 90% of the colistin-resistant and/or tigecycline-resistant strains. When the MIC distributions of ceftazidime-avibactam and aztreonam-avibactam were compared, of the 22 isolates resistant to ceftazidime-avibactam, only 18% (4/22) had high aztreonam-avibactam MICs (≥16 µg/mL). In contrast, all four isolates with high aztreonam-avibactam MICs were resistant to ceftazidime-avibactam (Supplemental Table S3).

**Figure 1:**
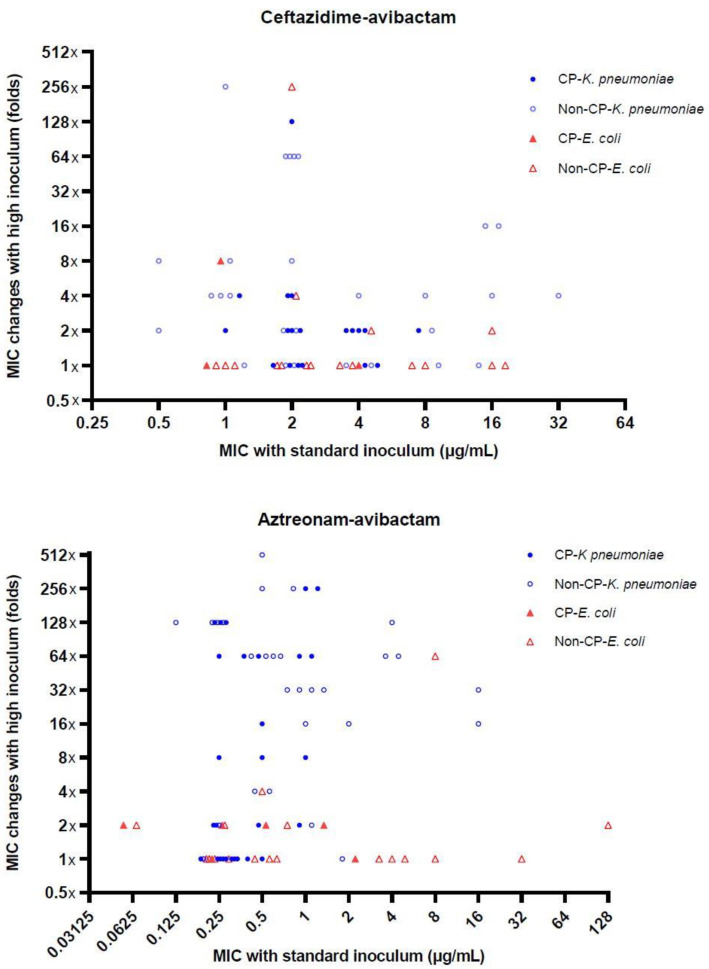
MIC changes (folds) with high inoculum for carbapenem-resistant *E. coli* and *K. pneumoniae* of (**top**) ceftazidime-avibactam and (**bottom**) aztreonam-avibactam.

When high inocula were used, the MIC_50_ of ceftazidime-avibactam increased from 4 to 8 μg/mL, and its MIC_90_ was ≥512 μg/mL, while those of aztreonam-avibactam increased from 0.5 to 4 μg/mL and from 4 to 256 μg/mL, respectively. Hence, 42% of CRE isolates became resistant to ceftazidime-avibactam with high inocula and 44% of the isolates exhibited high aztreonam-avibactam MICs (≥16 μg/mL) (Supplemental Table S1). The rates of the inoculum effect for ceftazidime-avibactam and aztreonam-avibactam were 18% and 47%, respectively (*p* < 0.001). *K. pneumoniae* isolates had a markedly higher rate of aztreonam-avibactam inoculum effects than *E. coli* (64% vs. 8%, *p* < 0.001) (Figure 1 and Table 2).

As shown in Table 3, the ceftazidime-avibactam MIC_50_ and MIC_90_ values against 46 non-CP-CRE isolates were 2 and 16 µg/mL, respectively. The ESBL test was positive in 80.4% (37/46) of these isolates, and AmpC β-lactamase in 19.6% (9/46). The ceftazidime-avibactam MIC_50_/MIC_90_ values of the CP-CRE isolates (n = 35) were 4/≥512 µg/mL, higher than those of the non-CP-CRE. Among the CP-CRE, isolates harboring NDM were mostly resistant to ceftazidime-avibactam. Unlike for ceftazidime-avibactam, the CP-CRE isolates exhibited lower MIC_50_/MIC_90_ values than the non-CP-CRE isolates for aztreonam-avibactam (0.25/1 µg/mL vs. 0.5/8 µg/mL) and there was no difference in MIC between isolates harboring KPC and NDM carbapenemases. The distribution of the resistance mechanisms of the study isolates, antimicrobial susceptibilities, and their rates of the inoculum effect stratified by resistance mechanism in each species, are shown in Supplemental Tables S4 and S5. The majority (72%) of carbapenem-resistant *E. coli* did not harbor carbapenemases, and most carried ESBLs. The inoculum effect for both ceftazidime-avibactam and aztreonam-avibactam was more common in CP *E. coli* isolates than non-CP *E. coli* isolates. In *K. pneumoniae*, non-CP isolates and CP isolates were evenly distributed, and KPC was the most prevalent (54%) carbapenemase. In contrast to *E. coli*, non-CP *K. pneumoniae* isolates were significantly more likely to show the inoculum effect for ceftazidime-avibactam and aztreonam-avibactam than CP *K. pneumoniae* isolates (*p* = 0.03 and *p* = 0.03, respectively).

## 4. Discussion

In the present study, ceftazidime-avibactam was active against 73% of CRE isolates, and aztreonam-avibactam had a low MIC (≤8 µg/mL) against 95% of the CRE isolates. In total, 43% of the study isolates were CP-CRE isolates, of which 34% harbored MBL. Unlike ceftazidime-avibactam, aztreonam-avibactam was less active against non-CP-CRE isolates than against CP-CRE isolates. The inoculum effect was more consistently detected with aztreonam-avibactam than with ceftazidime-avibactam, especially in *K. pneumoniae* isolates. To our knowledge, this is the first study to compare the in vitro activities of ceftazidime-avibactam and aztreonam-avibactam together with evaluating the inoculum effect against CRE isolates encompassing CP-CRE and non-CP CRE.

Our data suggest that aztreonam-avibactam may be more active than ceftazidime-avibactam against CRE strains. This result is similar to previous reports that found lower MIC_50_/MIC_90_ values for aztreonam-avibactam than for ceftazidime-avibactam against Gram-negative bacilli [24,25,26,27,28,29,30]. However, those studies included only CP-CRE, either alone or along with non-CRE. To date, ceftazidime-avibactam stands out as one of the most important additions to the antimicrobial armamentarium, as it is the first marketed fixed combination with activity against CRE, including those with the OXA and KPC enzymes [4,31]. Notably, avibactam cannot inhibit MBL, and nor can any other new β-lactamase inhibitor such as vaborbactam and relebactam [29,32]. Aztreonam, a monobactam, is stable to MBL [33]. In previous studies, aztreonam, in combination with ceftazidime-avibactam or avibactam, showed promising activity against MBL in *Enterobacterales* [7,34,35]. Since the Food and Drug Administration (FDA) granted Qualified Infectious Disease Product and Fast Track designation to aztreonam-avibactam for CRE infections in November 2019, further efforts to place aztreonam-avibactam in the right position to combat against CRE are critical. Recently, a Chinese study assessed the in vitro activities of ceftazidime-avibactam and aztreonam-avibactam against 58 CRE isolates, including both CP-CRE and non-CP-CRE [36]. In that study, the non-CP-CRE (n = 14) had lower aztreonam-avibactam MICs than the CP-CRE. However, in our hands, aztreonam-avibactam was less active against non-CP-CRE (n = 46), than CP-CRE. In addition, an aztreonam-avibactam inoculum effect was more common in the non-CP-CRE. Some differences in the species composition of study isolates and/or molecular epidemiology may have caused different results between the two studies. Further study of more CRE isolates is needed to confirm these findings. Our data showed that the *K. pneumoniae* isolates were more resistant to colistin or tigecycline than the *E. coli* isolates, but they tended to have lower MICs for both ceftazidime-avibactam and aztreonam-avibactam. A between-species comparison of antimicrobial activity could also be helpful in the management of CRE infection.

In this study, aztreonam-avibactam had a higher rate of inoculum effects than ceftazidime-avibactam (47% and 18%, respectively), particularly in *K. pneumoniae* isolates. Given the growing body of concern over the high mortality and rapid dissemination of CRE infection, it is surprising that no studies have explored the inoculum effects of ceftazidime-avibactam and aztreonam-avibactam—the two essential therapeutic options—in CRE isolates. Whether the inoculum effect is clinically significant remains debatable [8,37,38]. In the era of carbapenem-resistance driven in large by a broader β-lactamase repertoire, the bacterial inoculum can reduce the activity of antimicrobial agents, particularly for β-lactam/β-lactamase combination drugs. Based on our data, aztreonam-avibactam may fail in the treatment of high-inoculum infections caused by CRE. Considering this, the susceptibility breakpoint for aztreonam-avibactam against CRE should be set at <8 µg/mL, although more clinical evidence is needed.

Our study has several limitations. First, the possibility of resistance mechanisms other than carbapenemase co-existing in the CPE isolates were not examined. Thus, we did not assess the entire resistance mechanism-specific impact on antimicrobial susceptibility in an ideal manner. Second, of the members of the *Enterobacterales*, we selectively collected *E. coli* and *K. pneumoniae* species, and the species mainly harboring intrinsic AmpC β-lactamases were not included. Further studies of these species are warranted. Additionally, ceftazidime-avibactam and aztreonam-avibactam have not been in clinical use in South Korea so the absence of prior exposure to these antimicrobial agents may have affected the MIC values of these two agents and resistance rates. Despite these limitations, the in vitro activities of ceftazidime-avibactam and aztreonam-avibactam against the CRE isolates in our study are consistent with previous CP-CRE reports based on extensive epidemiologic data. Moreover, this study provided data for non-CP-CRE, for which there has been less reliable clinical data than for CP-CRE. Future studies to determine the optimal dosing and breakpoints of aztreonam-avibactam, and the benefit of combination therapy are warranted.

Ceftazidime-avibactam has been considered a reasonable option for the treatment of CRE infection. In this study, aztreonam-avibactam was more active against CRE than ceftazidime-avibactam, even for CP-CRE and ceftazidime-avibactam-resistant isolates. Currently available data may render aztreonam-avibactam a “game changer” in the treatment of difficult-to-treat Gram-negative organisms of various resistance mechanisms, including MBL. However, aztreonam-avibactam is not a one-size-fits-all option. The presence of the substantial inoculum effect may contribute to clinical failure in patients treated with aztreonam-avibactam for high inoculum CRE infections.

## Figures and Tables

**Table 1:** Antimicrobial susceptibility of carbapenem-resistant *E. coli* (n = 25) and *K. pneumoniae* (n = 56) isolates to seven antimicrobial agents.

Species	Antimicrobial Agent	Inoculum Size	Cumulative% of Isolates with Indicated MICs (μg/mL)														MIC (μg/mL)		S ^a^
			0.06	0.125	0.25	0.5	1	2	4	8	16	32	64	128	256	≥512	MIC_50_	MIC_90_	
**Non-CP-*E. coli* (18)**	CAZ	Standard						5.6				11.1		16.7	38.9	100	≥512	≥512	5.6
		High											5.6		11.1	100	≥512	≥512	0
	CAZ-AVI	Standard					16.7	50.0	66.7	77.8	94.4					100	2	16	77.8
		High					16.7	38.9	50.0	72.2	83.3	88.9				100	4	≥512	72.2
	ATM	Standard								5.6			22.2		27.8	100	≥512	≥512	0
		High											5.6		11.1	100	≥512	≥512	0
	ATM-AVI	Standard	5.6		33.3	55.6	61.1		77.8	88.9		94.4		100			0.5	32	NA ^b^
		High		5.6	27.8	50.0		61.1	77.8	83.3		88.9			94.4	100	0.5	256	NA
	MEM	Standard			5.6		16.7	22.2	27.8	66.7	88.9	100					8	32	16.7
		High				5.6	11.1	22.2	27.8	61.1	83.3	94.4	100				8	32	11.1
	CST	Standard			55.6	94.4			100								0.25	0.5	94.4
	TGC	Standard		11.1	38.9	66.7	83.3	88.9			94.4	100					0.5	16	66.7
**CP-*E. coli* (7)**	CAZ	Standard										14.3			28.6	100	≥512	≥512	0
		High													28.6	100	≥512	≥512	0
	CAZ-AVI	Standard					28.6		42.9							100	≥512	≥512	42.9
		High					14.3		28.6	42.9						100	≥512	≥512	42.9
	ATM	Standard					14.3	28.6						42.9	57.1	100	256	≥512	28.6
		High					14.3	28.6							42.9	100	≥512	≥512	28.6
	ATM-AVI	Standard	28.6		57.1	71.4	85.7	100									0.25	2	NA
		High		14.3	28.6	42.9	57.1	85.7				100					1	32	NA
	MEM	Standard						14.3		28.6	42.9		71.4	85.7	100 ^c^		64	256	0
		High									14.3	28.6	57.1		100 ^c^		32	≥256	0
	CST	Standard			14.3	100											0.5	0.5	100
	TGC	Standard			57.1	85.7			100								0.5	4	85.7
**Non-CP-*K. pneumoniae***	CAZ	Standard					3.6		7.1	10.7			14.3		32.1	100	≥512	≥512	7.1
		High					3.6			7.1				10.7		100	≥512	≥512	3.6
**(28)**	CAZ-AVI	Standard				7.1	28.6	60.7	71.4	82.1	96.4	100					2	≥512	82.1
		High					7.1	14.3	42.9	50.0	64.3	67.9	71.4	89.3	100		8	≥512	50.0
	ATM	Standard					10.7							14.3	17.9	100	≥512	≥512	10.7
		High					3.6				7.1			10.7		100	≥512	≥512	3.6
	ATM-AVI	Standard		3.6	21.4	50.0	75.0	82.1	92.9		100						0.5	4	NA
		High			13.6	7.1		21.4			28.6	71.4		75.0	92.9	100	32	256	NA
	MEM	Standard			7.1	17.9		21.4	28.6	42.9	75.0	92.9	100				16	32	17.9
		High				3.6		10.7	14.3	28.6	53.6	71.4	78.6	92.9	100 ^c^		16	128	3.6
	CST	Standard			14.3	64.3			67.9	75.0	85.7			96.4	100 ^c^		0.5	128	64.3
	TGC	Standard				7.1	42.9	64.3	89.3	96.4					100		1	8	7.1
**CP-*K. pneumoniae***	CAZ	Standard												32.1	57.1	100	≥512	≥512	0
		High													3.6	100	≥512	≥512	0
**(28)**	CAZ-AVI	Standard					7.1	42.9	64.3	67.9						100	4	≥512	67.9
		High						17.9	39.3	60.7	64.3				67.9	100	8	≥512	60.7
	ATM	Standard													17.9	100	≥512	≥512	0
		High													3.6	100	≥512	≥512	0
	ATM-AVI	Standard			53.6	78.6	100										0.25	1	NA
		High			28.6	42.9	46.4	53.6	57.1	64.3	67.9	85.7	92.9		100		2	64	NA
	MEM	Standard						3.6	7.1		10.7	25.0	53.6	64.3	100 ^c^		64	≥256	0
		High						3.6			7.1	10.7	25.0	46.4	100 ^c^		≥256	≥256	0
	CST	Standard			14.3	100											0.5	0.5	100
	TGC	Standard					17.9	42.9	75.0	92.9	96.4		100				4	8	0

CP, carbapenemase-producing; non-CP, non-carbapenemase-producing; MIC, minimum inhibitory concentration; CAZ, ceftazidime; CAZ-AVI, ceftazidime-avibactam; ATM, aztreonam; ATM-AVI, aztreonam-avibactam; MEM, meropenem; CST, colistin; TGC, tigecycline. ^a^ CLSI susceptibility breakpoints were used: ceftazidime, ≤4 μg/mL; ceftazidime-avibactam, ≤8/4 μg/mL; aztreonam, ≤4 μg/mL; meropenem, ≤1 μg/mL; 2019 EUCAST susceptibility breakpoints were used for colistin and tigecycline: colistin, ≤2 μg/mL; tigecycline, ≤0.5 μg/mL. ^b^ Not available because no breakpoint criteria have been defined for aztreonam-avibactam. ^c^ MIC is greater than or equal to the indicated value.

**Table 2:** Positive rates of inoculum effect for carbapenem-resistant isolates.

Antimicrobial Agent (Resistance Mechanism)	No. of Isolates (%) with Inoculum Effect ^a^			*p* Value
	Total	*E. coli*	*K. pneumoniae*	
**Ceftazidime-avibactam ^b^**	**12/67 (17.9)**	**2/20 (10)**	**10/47 (21.3)**	**0.27**
in CP-CRE	2/22 (9.1)	1/3 (33.3)	1/19 (5.3)	0.26
in non-CP-CRE	10/45 (22.2)	1/17 (5.9)	9/28 (32.1)	0.04
**Aztreonam-avibactam**	**38/81 (46.9)**	**2/25 (8.0)**	**36/56 (64.3)**	**<0.001**
in CP-CRE	15/35 (42.9)	1/7 (14.3)	14/28 (50)	0.10
in non-CP-CRE	23/46 (50)	1/18 (5.6)	22/28 (78.6)	<0.001

CP, carbapenemase-producing; CRE, carbapenem-resistant *Enterobacterales*; non-CP, non-carbapenemase-producing. ^a^ Inoculum effect was defined as an eightfold or greater increase in MIC on testing with the higher inoculum. ^b^ As the MIC values higher than 256 μg/mL for ceftazidime-avibactam were not further identified, the presence of the inoculum effect in the 14 isolates with these MICs was not determined.

**Table 3:** Antimicrobial susceptibility of carbapenem-resistant isolates according to resistance mechanism and inoculum.

Mechanism (n)	Antimicrobial Agent	Inoculum Size	Cumulative% with Indicated MICs (μg/mL)														MIC (μg/mL)		S ^a^
			0.06	0.125	0.25	0.5	1	2	4	8	16	32	64	128	256	≥512	MIC_50_	MIC_90_	
**Non-CP-CRE** **(46) ^b^**	**CAZ-AVI**	**Standard**				4.3	23.9	56.5	69.6	80.4	95.7	97.8				100	2	16	80.4
		High					10.9	23.9	45.7	58.7	71.7	76.1	78.3	89.1	95.7	100	8	256	58.7
	ATM-AVI	Standard	2.2	4.3	26.1	52.2	69.6	73.9	87.0	91.3	95.7	97.8		100			0.5	8	NA ^c^
		High		2.2	13	23.9		37	43.5	45.7	50	78.3		80.4	93.5	100	16	256	NA
ESBL (30)	CAZ-AVI	Standard				6.7	16.7	50.0	63.3	80.0	93.3	96.7				100	2	16	80.0
		High					10.0	23.3	33.3	53.3	66.7	73.3	76.7	90.0	93.3	100	8	128	53.3
	ATM-AVI	Standard	3.3	6.7	30.0	60.0	73.3		86.7	93.3	100						0.5	8	NA
		High		3.3	16.7	30		33.3	40	43.3	50	73.3		76.7	93.3	100	16	256	NA
AmpC (2)	CAZ-AVI	Standard					100										-	-	100
		High					50.0								100		-	-	50.0
	ATM-AVI	Standard				50.0	100										-	-	NA
		High						50.0		‘		100					-	-	NA
ESBL + AmpC (7)	CAZ-AVI	Standard					28.6	85.7	100								2	4	100
		High						14.3	85.7					100			4	128	
	ATM-AVI	Standard			14.3	42.9	71.4	100									1	2	NA
		High						42.9				100					32	32	NA
**CP-CRE** **(35)**	CAZ-AVI	Standard					11.4	40.0	60.0	62.9						100	4	≥512	62.9
		High					2.9	17.1	37.1	57.1	60.0				62.9	100	8	≥512	57.1
	ATM-AVI	Standard	5.7		54.3	77.1	97.1	100									0.25	1	NA
		High		2.9	28.6	42.9	48.6	60.0	62.9	68.6	71.4	88.6	94.3		100		2	64	NA
KPC (17)	CAZ-AVI	Standard					11.8	58.8	82.4							100	2	≥512	82.4
		High						11.8	41.2	76.5					82.4	100	8	≥512	76.5
	ATM-AVI	Standard	5.9		58.8	82.4	100										0.25	1	NA
		High			35.3	47.1	58.8	64.7		70.6		94.1			100		1	32	NA
NDM (11)	CAZ-AVI	Standard						9.1	18.2	27.3						100	≥512	≥512	27.3
		High						9.1	18.2		27.3					100	≥512	≥512	18.2
	ATM-AVI	Standard	9.1		54.5	63.6	90.9	100									0.25	1	NA
		High		9.1	36.4	54.5		81.8					100				0.5	64	NA

MIC, minimum inhibitory concentration; Non-CP CRE, non-carbapenemase-producing carbapenem-resistant *Enterobacterales*; ESBL, extended-spectrum beta-lactamase; AmpC, AmpC beta-lactamase; CAZ, ceftazidime; CAZ-AVI, ceftazidime-avibactam; ATM, aztreonam; ATM-AVI, aztreonam-avibactam; MEM, meropenem; CST, colistin; TGC, tigecycline. ^a^ CLSI susceptibility breakpoints were used: ceftazidime, ≤4 μg/mL; ceftazidime-avibactam, ≤8/4 μg/mL; aztreonam, ≤4 μg/mL; meropenem, ≤1 μg/mL; 2019 EUCAST susceptibility breakpoints were used for colistin and tigecycline: colistin, ≤2 μg/mL; tigecycline, ≤0.5 μg/mL. ^b^ Four *E. coli* isolates and three *K. pneumoniae* isolates did not produce ESBL nor AmpC beta-lactamases, respectively. ^c^ Not available because no breakpoint criteria have been defined for aztreonam-avibactam.

## Supplementary Materials

The following are available online at https://www.mdpi.com/2079-6382/9/12/912/s1, Table S1: Antimicrobial susceptibility of carbapenem-resistant *E. coli* and *K. pneumoniae* isolates to seven antimicrobial agents, Table S2: Ceftazidime-avibactam and aztreonam-avibactam MIC distribution according to meropenem, colistin and tigecycline susceptibility pattern in carbapenem-resistant isolates, Table S3: MIC distributions of ceftazidime-avibactam and aztreonam-avibactam for carbapenem-resistant *E. coli* and *K. pneumoniae*, Table S4: Resistance mechanisms of carbapenem-resistant *E. coli* (n = 25) and *K. pneumoniae*, Table S5: Antimicrobial susceptibility and positive rate of inoculum effect of carbapenem-resistant isolates according to resistance mechanism.
