# Supplementary material for: In Vitro Activities and Inoculum Effects of Ceftazidime-Avibactam and Aztreonam-Avibactam against Carbapenem-Resistant Enterobacterales Isolates from South Korea
Source: Antibiotics (Basel). 2020 Dec 15;9(12):912. doi: 10.3390/antibiotics9120912 (PMC7765481; doi:10.3390/antibiotics9120912)
Supplement: Supplementary file 1 [file antibiotics-09-00912-s001.pdf]

antibiotics

Article

In Vitro Activities and Inoculum Effects of eftazidime-Avibactam and Aztreonam-Avibactam against Carbapenem-Resistant *Enterobacterales* Isolates from South Korea

Taeeun Kim [1], Seung Cheol Lee [2], Moonsuk Bae [2], Heungsup Sung [3], Mi-Na Kim [3], Jiwon Jung [2], Min Jae Kim [2], Sung-Han Kim [2], Sang-Oh Lee [2], Sang-Ho Choi [2], Yang Soo Kim [2] and Yong Pil Chong [2,*]

[1] Division of Infectious Diseases, Department of Medicine, Nowon Eulji University Hospital, Seoul 01830, Korea; sleepju@naver.com
[2] Department of Infectious Diseases, Asan Medical Center, University of Ulsan College of Medicine, Seoul 05505, Korea; sclee628@naver.com (S.C.L.); carukeion@gmail.com (M.B.); trueblue27@naver.com (J.J.); nahani99@gmail.com (M.J.K.); kimsunghanmd@hotmail.com (S.-H.K.); soleemd@amc.seoul.kr (S.-O.L.); sangho@amc.seoul.kr (S.-H.C.); yskim@amc.seoul.kr (Y.S.K.)
[3] Department of Laboratory Medicine, Asan Medical Center, University of Ulsan College of Medicine, Seoul 05505, Korea; sung@amc.seoul.kr (H.S.); mnkim@amc.seoul.kr (M.-N.K.)
* Correspondence: drchong@amc.seoul.kr; Tel.: +82-2-3010-3306; Fax: +82-3010-3306

Supplemental Table S1. Antimicrobial susceptibility of carbapenem-resistant *E. coli* and *K. pneumoniae* isolates to seven antimicrobial agents (n=81).

Antimicrobial agent	Inoculum size	No. of isolates (cumulative %) with indicated MICs (µg/mL)														MIC (µg/mL)		S
		0.06	0.125	0.25	0.5	1	2	4	8	16	32	64	128	256	≥512	MIC_{50}	MIC_{90}	n (%)[a]
**CAZ**	**Standard**					1 (1.2)	1 (2.5)	1 (3.7)	1 (4.9)		2 (7.4)	1 (8.6)	10 (21.0)	17 (42.0)	47 (100)	≥512	≥512	3 (3.7)
	High					1 (1.2)			1 (2.4)			1 (3.6)	1 (4.8)	4 (9.9)	73 (100)	≥512	≥512	1 (1.2)
CAZ-AVI	Standard				2 (2.5)	13 (18.5)	25 (49.4)	13 (65.4)	6 (72.8)	7 (81.5)	1 (82.7)				14 (100)	4	≥512	59 (72.8)
	High					6 (7.4)	11 (21.0)	17 (42.0)	13 (58.0)	17 (66.7)	2 (69.1)	1 (70.4)	5 (76.5)	4 (81.5)	15 (100)	8	≥512	47 (58.0)
ATM	Standard					4 (4.9)	1 (6.2)		1 (7.4)			3 (11.1)	2 (13.6)	8 (23.5)	62 (100)	≥512	≥512	5 (6.2)
	High					2 (2.5)	1 (3.7)			1 (4.9)		1 (6.2)	1 (7.4)	3 (11.1)	72 (100)	≥512	≥512	3 (3.7)
ATM-AVI	Standard	3 (3.7)	1 (4.9)	27 (38.3)	20 (63.0)	15 (81.5)	3 (85.2)	6 (92.6)	2 (95.1)	2 (97.5)	1 (98.8)		1 (100)			0.5	4	NA[b]
	High		2 (2.5)	14 (19.8)	10 (31.1)	2 (34.6)	10 (46.9)	4 (51.9)	3 (55.6)	3 (59.3)	19 (82.7)	2 (85.2)	1 (86.4)	8 (96.3)	3 (100)	4	256	NA[b]
MEM	Standard			3 (3.7)	3 (7.4)	2 (9.9)	4 (14.8)	4 (19.8)	12 (34.6)	15 (53.1)	11 (66.7)	12 (81.5)	4 (86.4)	11[c] (100)		16	≥256	8 (9.9)
	High				2 (2.5)	1 (3.7)	5 (9.9)	2 (12.3)	10 (24.7)	13 (40.7)	9 (51.9)	9 (63.0)	10 (75.3)	20[c] (100)		32	≥256	3 (3.7)
CST	Standard			19 (23.5)	51 (86.4)			2 (88.9)	2 (91.4)	3 (95.1)			3 (98.8)	1[c] (100)		0.5	8	70 (86.4)
TGC	Standard		2 (2.5)	9 (13.6)	9 (24.7)	18 (46.9)	14 (64.2)	17 (85.2)	7 (93.8)	2 (96.3)	1 (97.5)	1 (98.8)		1 (100)		2	8	20 (24.7)

MIC, minimum inhibitory concentration; CAZ, ceftazidime; CAZ-AVI, ceftazidime-avibactam; ATM, aztreonam; ATM-AVI, aztreonam-avibactam; MEM, meropenem; CST, colistin; TGC, tigecycline; NA, not available.[a] CLSI susceptibility breakpoints were used: ceftazidime, ≤4 µg/mL; ceftazidime-avibactam, ≤8/4 µg/mL; aztreonam, ≤4 µg/mL; meropenem, ≤1µg/mL; 2019 EUCAST susceptibility breakpoints were used for colistin and tigecycline: colistin, ≤2 µg/mL; tigecycline, ≤0.5 µg/mL.[b] Not available because no breakpoint criteria have been defined for aztreonam-avibactam.[c] MIC is greater than or equal to the indicated value.

Supplemental Table S2. Ceftazidime-avibactam and aztreonam-avibactam MIC distribution according to meropenem, colistin and tigecycline susceptibility pattern in carbapenem-resistant isolates.

	**Colistin susceptibility[b]**		P value	Tigecycline susceptibility[c]		P value
	**Susceptible (n = 70)**	Resistant (n = 11)		Susceptible (n = 20)	Resistant (n = 61)	
Ceftazidime-avibactam MIC[a]						
S (≤8 µg/mL)	50 (71.4)	9 (81.8)	.47	14 (70.0)	45 (73.8)	.74
R (>8 µg/mL)	20 (28.6)	2 (18.2)		6 (30.0)	16 (26.2)	
Aztreonam-avibactam MIC						
Lower MIC (≤8 µg/mL)	67 (95.7)	10 (90.9)	.50	19 (95.0)	58 (95.1)	.99
Higher MIC (>8 µg/mL)	3 (4.3)	1 (9.1)		1 (5.0)	3 (4.9)	

Data are presented as the number of isolates with the corresponding percentage shown in parentheses. [a] Ceftazidime-avibactam CLSI breakpoints: S ≤8/4 µg/mL, R >8/4 µg/mL. [b] Colistin EUCAST breakpoints: S ≤2 µg/mL, R >2 µg/mL. [c] Tigecycline EUCAST breakpoints: S ≤0.5 µg/mL, R >0.5 µg/mL.

Supplemental Table S3. MIC distributions of ceftazidime-avibactam and aztreonam-avibactam for carbapenem-resistant *E. coli* and *K. pneumoniae* (n=81).

**AZT/AVI, MIC (µg/mL)**	**CAZ-AVI MIC (µg/mL) No. of isolates (no. of CPE) with indicated MIC**											**Grand total (No. of CPE)**
	0.5	1	2	4	8	**16**	32	64	128	256	>256	
0.06		1 (1)									2 (1)	3 (2)
0.125	1 (0)											1 (0)
0.25	1 (0)	8 (3)	9 (6)		1 (0)						8 (8)	27 (17)
0.5		3 (0)	9 (4)	6 (3)	1 (0)						1 (1)	20 (8)
1		1 (0)	4 (0)	5 (4)	1 (1)	2 (0)					2 (2)	15 (7)
2			1 (0)	1 (0)							1 (1)	3 (1)
4			1 (0)	1 (0)	3 (0)	1 (0)						6 (0)
8			1 (0)			1 (0)						2 (0)
**16**						1 (0)	1 (0)					2 (0)
32						1 (0)						1 (0)
64												–
128						1 (0)						1 (0)
**Grand total (No. of CPE)**	2 (0)	13 (4)	25 (10)	13 (7)	6 (1)	7 (0)	1 (0)	–	–	–	14 (13)	81

Supplemental Table S4. Resistance mechanisms of carbapenem-resistant *E. coli* (n=25) and *K. pneumoniae* (n=56).

Mechanism	*E. coli*, n (%)	*K. pneumoniae*, n (%)	Total, n (%)
**Non-CP-CRE**	18 (72.0)	28 (50.0)	46 (100)
ESBL	13 (52.0)	17 (30.4)	30 (65.2)
AmpC	1 (4.0)	1 (1.8)	2 (4.3)
ESBL+AmpC	0	7 (12.5)	7 (15.2)
Others	4 (16.0)	3 (5.4)	7 (15.2)
**CP-CRE**	7 (28.0)	28 (50.0)	35 (100)
KPC	2 (8.0)	15 (26.8)	17 (48.6)
NDM	4 (16.0)	7 (12.5)	11 (31.4)
OXA-48-like	1 (4.0)	3 (5.4)	4 (11.4)
VIM	0	1 (1.8)	1 (2.9)
Others	0	2 (3.6)	2 (5.7)

Data are presented as the number of isolates with the corresponding percentage shown in parentheses. Non-CP-CRE, non-carbapenemase-producing carbapenem-resistant Enterobacteriaceae. .

Supplemental Table S5. Antimicrobial susceptibility and positive rate of inoculum effect of carbapenem-resistant isolates according to resistance mechanism.

Species (n)	Mechanism (n)	Antimicrobial agent	Inoculum size	MIC (μg/mL)			S n (%)[a]	No. of isolates (%) with inoculum effect
				**MIC50**	MIC90	Range		
***E. coli* (25)**	**Non-CP CRE (18)**	**CAZ-AVI**	**Standard**	**2**	16	1 to ≥512	14 (77.8)	1/17 (5.9)
			High	4	≥512	1 to ≥512	13 (72.2)	
		ATM-AVI	Standard	0.5	32	0.06 to 128	NA	1/18 (5.6)
			High	0.5	256	0.125 to ≥512	NA	
	ESBL (13)	CAZ-AVI	Standard	2	16	1 to ≥512	11 (84.6)	1/12 (8.3)
			High	8	≥512	1 to ≥512	10 (76.9)	
		ATM-AVI	Standard	0.5	8	0.06 to 8	NA	1/13 (7.7)
			High	0.5	8	0.125 to 8	NA	
	AmpC (1)	CAZ-AVI	Standard	-	-	1	1 (100)	0
			High	-	-	1	1 (100)	
		ATM-AVI	Standard	-	-	0.5	NA	0
			High	-	-	2	NA	
	**CPE** (7)	CAZ-AVI	Standard	≥512	≥512	1 to ≥512	3 (42.9)	1/3 (33.3)
			High	≥512	≥512	1 to ≥512		
		ATM-AVI	Standard	0.25	2	0.06 to 2	NA	1/7 (14.3)
			High	1	32	0.125 to 2	NA	
	KPC (2)	CAZ-AVI	Standard	-	-	1 to 4	2 (100)	1/2 (50.0)
			High	-	-	4 to 8	2 (100)	
		ATM-AVI	Standard	-	-	0.5 to 1	NA	1/2 (50.0)
			High	-	-	0.06 to 32	NA	
	NDM (4)	CAZ-AVI	Standard	≥512	≥512	≥512	0	ND
			High	≥512	≥512	≥512	0	
		ATM-AVI	Standard	0.25	2	0.06 to 2	NA	0
			High	0.25	2	0.124 to 2	NA	
K. pneumoniae (56)	**Non-CP CRE** (28)	CAZ-AVI	Standard	2	16	0.5 to 32	23 (82.1)	9/28 (32.1)
			High	8	256	1 to 256	14 (50.0)	
		ATM-AVI	Standard	0.5	4	0.125 to 16	NA	22/28 (78.6)
			High	32	256	0.25 to ≥512	NA	
	ESBL (17)	CAZ-AVI	Standard	4	16	0.5 to 32	13 (76.5)	6/17 (35.3)
			High	16	128	1 to 256	6 (35.3)	
		ATM-AVI	Standard	0.5	16	0.125 to 16	NA	16/17 (94.1)
			High	32	256	2 to ≥512	NA	
	AmpC (1)	CAZ-AVI	Standard	-	-	1	1 (100)	1/1 (100)
			High	-	-	256	0	
		ATM-AVI	Standard	-	-	1	NA	1/1 (100)
			High	-	-	32	NA	
	ESBL +AmpC (7)	CAZ-AVI	Standard	2	4	1 to 4	7 (100)	1/7 (14.3)
			High	4	128	2 to 128	6 (85.7)	
		ATM-AVI	Standard	1	2	0.25 to 2	NA	4/7 (57.1)
			High	32	32	2 to 32	NA	
	**CPE** (28)	CAZ-AVI	Standard	4	≥512	1 to ≥512	19 (67.9)	1/19 (5.3)
			High	8	≥512	2 to ≥512	17 (60.7)	
		ATM-AVI	Standard	0.25	1	0.25 to 1	NA	14/28 (50.0)
			High	2	64	0.25 to 256	NA	
	KPC (15)	CAZ-AVI	Standard	2	≥512	2 to ≥512	12 (80.0)	1/12 (8.3)
			High	8	≥512	8 to ≥512	11 (73.3)	
		ATM-AVI	Standard	0.25	1	0.25 to 1	NA	5/15 (33.3)
			High	0.5	32	0.25 to 256	NA	
	NDM (7)	CAZ-AVI	Standard	≥512	≥512	2 to ≥512	3 (42.9)	0/3
			High	≥512	≥512	2 to ≥512	2 (28.6)	
		ATM-AVI	Standard	0.25	1	0.25 to 1	NA	3/7 (42.9)
			High	0.5	64	0.25 to 64	NA	

Non-CP-CRE, non-carbapenemase-producing carbapenem-resistant Enterobacteriaceae; CAZ-AVI, ceftazidime-avibactam; ATM-AVI, aztreonam-avibactam, NA, not available; ND, not determined.[a] CLSI susceptibility breakpoints were used: ceftazidime, ≤4 μg/mL; ceftazidime-avibactam,

≤8/4 μg/mL; aztreonam, ≤4 μg/mL; meropenem, ≤1μg/mL; 2019 EUCAST susceptibility breakpoints were used for colistin and tigecycline: colistin, ≤2 μg/mL; tigecycline, ≤0.5 μg/mL.
